# Insights from critical care nurses: role of leadership coaching in enhancing incident reporting culture: a cross-sectional study

**DOI:** 10.1186/s12912-025-03791-6

**Published:** 2025-09-02

**Authors:** Boshra Karem Mohamed El-Sayed, Eman Asad Taha Mohmed, Fatma Mostafa Baddar, Sabrein Mahmoud Khalifa Khattab

**Affiliations:** 1 Nursing Administration Department, Faculty of Nursing, Alexandria University, Alexandria, Egypt; 2 Neonatal Intensive Care Unit at El Maternity Hospital, Alexandria, Egypt

**Keywords:** Critical care nurses, Leadership coaching behavior, Incident reporting culture, Patient safety

## Abstract

**Background:**

Incident reporting in critical care settings is essential for patient safety, yet underreporting remains a challenge. Leadership coaching has emerged as a potential strategy to enhance a culture of safety by empowering nurses to actively engage in incident reporting.

**Aim:**

This study explores the role of leadership coaching behavior in incident reporting culture among critical care nurses and investigates the relationship between leadership coaching behavior and various subdomains of incident reporting culture among nurses in critical care units.

**Methods:**

A cross-sectional study was conducted at all critical care units of Alexandria Main University Hospital, Egypt. Data were collected from a convenience sample of 240 critical care nurses via the Incident Reporting Culture Questionnaire and Leader Coaching Behavior Questionnaire. Correlation and regression analyses were utilized to achieve the aims of the study.

**Results:**

Critical care nurses reported moderate levels of leadership coaching behavior (3.65 ± 0.82) and incident reporting culture (3.23 ± 0.50). Leadership coaching behavior correlated strongly with incident reporting culture (*r* = 0.617, *p* < 0.001), particularly in terms of communication, learning from errors, and feedback provision. Hierarchical regression revealed that adding leadership coaching behavior increased the explained variance from 23.5 to 50.5% (ΔR² = 0.270, *p* < 0.001), making it the strongest predictor.

**Conclusions:**

The findings highlight the critical role of leadership coaching behavior in fostering an open and supportive incident reporting culture in critical care settings. Integrating coaching strategies into leadership development programs may enhance patient safety by encouraging transparency and accountability among nursing staff.

**Relevance to clinical practice:**

Nurse managers should design coaching programs to empower nurses to identify and report safety concerns more effectively, resulting in more comprehensive data collection and enhanced risk management. Additionally, leadership coaching can be integrated into hospital policies and professional development programs, ensuring a long-term commitment to improving safety culture. Nursing schools can employ real-world scenarios to teach students how to navigate reporting systems and address patient safety concerns effectively.

**Clinical trial number:**

Not applicable.

## Introduction

Patient safety remains a central concern in healthcare, particularly within critical care environments where high patient acuity, rapid clinical decision-making, and technological complexity increase the risk of adverse events. Incident reporting is a fundamental mechanism for identifying system failures and driving improvements; however, ongoing underreporting, especially in critical care units (CCUs), undermines these efforts [1, 2]. The contributing factors include fear of blame, punitive institutional responses, and organizational cultures that deprioritize transparency in favor of operational efficiency, leaving critical safety vulnerabilities unaddressed [3, 4].

Leadership is decisive in influencing reporting behaviors and shaping the broader safety culture within healthcare organizations. Traditional hierarchical leadership structures often perpetuate environments of silence and blame, discouraging staff from disclosing errors or near misses [5, 6]. In contrast, leadership coaching provides a transformative approach by fostering psychological safety, enhancing communication, and promoting a learning-oriented response to incidents among critical care nurses (CCNs). This contemporary leadership model has the potential to significantly improve incident reporting practices and strengthen patient safety outcomes in high-risk clinical settings [7, 8].

## Background

An incident reporting culture is an organizational framework that promotes the open, timely, and non-punitive reporting of errors, near misses, and safety concerns to improve overall safety and quality [9]. This culture is characterized by transparency, leadership support, effective communication channels, and robust feedback systems. Research shows that when organizations prioritize a strong reporting culture, they experience improved identification of risks, reduced recurrence of similar incidents, and increased staff engagement in safety practices [10]. In healthcare, a positive incident reporting culture has been linked to better patient outcomes and lower rates of adverse events. Effective systems that support open communication, learn from errors, and empower nurses to share concerns without fear lead to proactive risk management and continuous improvement [11, 12]. Studies also indicate that organizations with well-established feedback mechanisms are more successful in fostering trust and accountability, which are essential for sustaining a culture of safety [13].

Despite the recognized importance of incident reporting, several persistent barriers continue to hinder its effectiveness. These include fear of punitive consequences, time constraints due to heavy workloads, insufficient training, and weak feedback mechanisms [2]. In a qualitative study conducted in South Africa, Gqaleni and Mkhize (2024) reported that inadequate institutional support, insufficient education and training, and poor human resources were major barriers to implementing patient safety incident reporting and learning guidelines in specialized care units [5]. Furthermore, a study by Dhamanti et al. (2020) highlighted that the absence of feedback following incident reports significantly discourages staff from reporting future events, thereby impeding organizational learning and safety improvements [14]. These findings underscore the need for healthcare organizations to address these barriers by fostering a nonpunitive culture, streamlining reporting processes, providing adequate training, and ensuring timely feedback to encourage effective incident reporting [15].

A pivotal component in this endeavor is the role of leadership coaching. Effective leadership coaching can cultivate a culture of psychological safety, where staff feels empowered to report incidents without fear of retribution [16]. For instance, a study published in the *Journal of Patient Safety* emphasized that hierarchical cultures and poor leadership can undermine staff’s ability to recognize deterioration and escalate concerns effectively [17]. Leadership coaching has emerged as a transformative approach to developing healthcare leaders who can foster cultures of safety, learning, and continuous improvement. Unlike traditional training programs that focus on knowledge transfer, leadership coaching emphasizes skill development, self-awareness, and behavioral change through individualized support and guided reflection [18].

Recent evidence suggests that leadership coaching interventions can significantly influence organizational culture and safety outcomes. For example, a coaching programme implemented at the Medical University of South Carolina (MUSC) Health demonstrated notable improvements in employee engagement, safety culture, and patient experience. The program included training existing team members as certified coaches and assisting leaders in action planning and development. After a year, departments that participated in coaching showed significant enhancements in these areas, highlighting the effectiveness of leadership coaching in fostering a positive safety culture [19].

Leader coaching behavior in healthcare encompasses several key dimensions that collectively enhance team performance, safety culture, and staff satisfaction. A study by Calpbinici et al. (2024) identified four primary dimensions of coaching leadership: effective communication, the ability to give and receive feedback, delegation of authority and influence, and support of the team to achieve organizational goals [20]. These dimensions were positively correlated with improved safety climate and job satisfaction among nursing staff. Further research by Al-Oweidat et al. (2023) emphasized that leadership behaviors, including empowerment and supportive communication, significantly influence nurses’ incident reporting practices, thereby fostering a culture of safety [21]. Additionally, a study by Sobhy & Mahdy (2024) demonstrated the importance of coaching leadership in fostering an ethical work environment that encourages transparent communication and proactive error reporting, thereby contributing to improved patient safety outcomes [22]. Collectively, these studies underscore the multifaceted nature of leader coaching behavior and its critical role in promoting a positive and safe healthcare environment.

### Research gap and significance of the study

Despite the recognized importance of incident reporting in enhancing patient safety, there is a limited body of research examining the specific role of leadership coaching in fostering an effective reporting culture among critical care nurses. While studies have explored the influence of leadership styles and organizational culture on reporting behaviors, few have focused on how structured leadership coaching, an approach that emphasizes developmental feedback, trust-building, and empowerment, can help overcome barriers such as fear of retaliation and lack of psychological safety [21]. This gap is particularly concerning in CCUs, where the complexity and acuity of care increase the need for timely and transparent communication of incidents.

This study is significant because it addresses a high-risk area of nursing practice, critical care, where the potential consequences of unreported errors are particularly severe. Research has shown that leadership behaviors influence nurses’ willingness to report incidents, yet the application of leadership coaching in this context remains underexplored [21, 22]. By examining the relationship between leadership coaching and incident reporting culture in critical care settings, this study provides valuable insights into how leaders can create psychologically safe environments that encourage reporting, foster learning from errors, and ultimately improve patient safety outcomes.

The findings of this study are expected to inform hospital policies and professional development programs by highlighting the importance of coaching-based leadership in CCUs. This focus is particularly relevant given the evidence that nurse leaders who adopt supportive, coaching-oriented behaviors positively influence ethical awareness, accountability, and self-monitoring [22]. Moreover, the study fills a critical gap in the literature by offering empirical evidence on the effectiveness of leadership coaching as a tool for cultivating a robust incident reporting culture, thereby advancing both practice and research in healthcare leadership and patient safety.

### Theoretical foundations

The theoretical foundation of this study is grounded in the integration of psychological safety theory and the leadership coaching model, both of which are essential for understanding how leadership behaviors influence incident reporting in critical care settings. Psychological safety, originally conceptualized by Edmondson & Lei (2014), refers to an environment where individuals feel safe to voice concerns, admit mistakes, and report errors without fear of blame or retaliation [23]. Recent research by Pfeifer et al. (2023) confirmed that psychological safety significantly predicts nurses’ willingness to report safety events, particularly in high-stakes environments such as pediatric and critical care units. This underscores the importance of fostering a climate where transparency and learning from mistakes are encouraged to enhance patient safety outcomes [24].

Similarly, the leadership coaching model emphasizes developmental, trust-based interactions between leaders and staff. Coaching-based leadership promotes reflection and ethical awareness and provides key drivers of psychological safety. Hu et al. (2024) highlighted that leadership coaching improves staff confidence and communication, enabling nurses to engage more proactively in safety practices [25]. Similarly, Gqaleni et al. (2024) reported that leadership coaching in ICU settings enhanced team communication and increased the likelihood of incident reporting [26]. By integrating these two frameworks, this study provides a robust theoretical basis for exploring how leadership coaching can be leveraged to cultivate a safety-oriented culture among critical care nurses.

### Aim of the study

This study aims to assess the levels of perception of leadership coaching and incident reporting culture among CCNs. Furthermore, it aims to identify the correlations between these perceptions and analyze how leadership coaching influences the culture of incident reporting within critical care units (CCUs).

### Research question

What is the relationship between leadership coaching and incident reporting culture as perceived by CCNs?

To what extent does leadership coaching behavior predict incident reporting culture among critical care nurses after controlling for demographic and work-related factors?

## Materials and methods

### Research design

A cross-sectional exploratory research design was adopted for this study, enabling researchers to examine critical care nurses’ perceptions of leadership coaching behavior and incident reporting culture as they existed at a particular instance. This design is advantageous for identifying correlations and providing current insights efficiently, without requiring longitudinal data collection. All study procedures and reporting complied with the Strengthening the Reporting of Observational Studies in Epidemiology (STROBE) guidelines.

### Settings

The study was conducted across all critical care units at the Main University Hospital, the largest educational hospital in Alexandria, Egypt. The hospital provides public, nonpaid healthcare services and has a total bed capacity of over 6,760 beds, with 1,126 beds allocated to the 23 units providing critical care for adult patients. It is considered the largest educational university hospital in Alexandria and the first university hospital to make significant progress in meeting the requirements of the General Authority for Health Accreditation and Regulation (GAHAR) regarding patient safety.

### Participants and sampling

The study population employed critical care nurses working in the abovementioned hospital. The inclusion criteria were critical care nurses who had at least one year of work experience, were working full-time, provided direct care to patients, and agreed to participate in the study. To determine the appropriate sample size, the researchers used G*Power version 3.1.9.7 for linear multiple regression. The power analysis indicated that a sample size of 240 nurses would be needed to detect a small effect size of 0.05 for the F test, with a power of 80% and a type 1 error rate of 0.05.

The sample of 240 nurses was selected via a convenience sampling method (impact generalizability) from a total population of 310 nurses in the study setting. To account for potential nonresponse or attrition, the survey was distributed to all 310 eligible nurses, and 240 nurses participated, achieving the sample size needed.

### Study instruments

The study utilized three instruments to gather data. The nurses’ profile questionnaire, developed by the researchers, captured demographic and professional information, including age, sex, marital status, educational level, years of nursing experience in the nursing profession, years of experience in the current unit, and attendance of training programs related to different patterns, including leader coaching behavior and the reporting of unexpected incidents. The responses are presented as frequencies and percentages.

**Tool (I): The Incident Reporting Culture Questionnaire (IRCQ**) was developed by Chiang et al. (2011) to assess the level of perception of nurses toward incident reporting culture. It consists of 20 items classified into four dimensions, namely, the application of learning from errors (5 items), readiness to provide feedback on incident reports (6 items), and collegial atmospheres of unpleasantness and punishment (5 items), which are negative phrases and have reversed scores: incident management: confidential and system driven (4 items). Nurses were rated on a 5-point Likert scale ranging from (1) strongly disagree to (5) strongly agree. The overall score ranges from 20 to 100. A higher total score indicates a greater perception of incident reporting culture among nurses [27]. The reliability of the tool was tested by Chiang et al. (2020), and the results revealed high reliability, as the Cronbach’s alpha coefficient was 0.88 [28].

**Tool (II): The leader coaching behavior questionnaire (LCBQ)** developed by Cardoso et al. (2014) was used to assess nurses’ perceptions of leader coaching behavior. It consists of 20 items across four domains, namely, communication (5 items), giving and receiving feedback (5 items), delegating power and exerting influence (5 items) and supporting the team in achieving organizational results (5 items). Nurses’ responses were measured on a 5-point Likert scale ranging from (1) never to (5) always. The overall score ranges from 20 to 100. A higher total score indicates a greater perception of leader coaching behaviors among nurses [29]. The reliability of the scale was confirmed, with a Cronbach’s alpha coefficient of 0.84 [30].

### Study instrument validation and reliability

#### Tool translation

The study tools were translated into Arabic and then back into English via the back-to-back translation technique to ensure cultural relevance and mitigate potential threats to the study’s validity.

#### Content validity

After the instruments were translated, their validity was reviewed by five academic experts from the Faculty of Nursing, Alexandria University, to assess content validity. The wording suggested by the expert panel was modified on the basis of their recommendations and feedback. A pilot study was conducted with 10% (*n* = 24) of critical care nurses to evaluate item clarity and estimate the time required to complete the tools. The feedback indicated that the items were clear, and these nurses were excluded from the final sample.

#### Construct validity

To assess the construct validity of the translated scales, confirmatory factor analysis (CFA) was performed via AMOS V.28 for SPSS. An acceptable model fit was defined by a chi square (X²)/DF < 5, a goodness-of-fit index (GFI), an incremental fit index (IFI) and a comparative fit index (CFI) > 0.90 and a root mean square error of approximation (RMSEA) < 0.08 (West et al., 2023). On the basis of these criteria, the CFA for the IRCQ questionnaire demonstrated good model fit (CFI = 1.000, IFI = 1.000, RMSEA = 0.066, X² =4.523, *P* < 0.001). Similarly, the CFA for the LCBQ indicated strong model fit (CFI = 1.000, IFI = 1.000, RMSEA = 0.045, X² =3.060, *P* < 0.001).

### Reliability analysis

A reliability analysis was employed to assess the internal consistency of the indicators of the underlying factor. The reliability of the study instruments was assessed via Cronbach’s alpha. The IRCQ and LCBQ had overall coefficient alpha values of 0.803 and 0.945, respectively, indicating strong internal consistency for both instruments.

### Data collection

The data collection process involved distributing hand-delivered questionnaires to staff nurses across different categories during morning, evening, and night shifts. Each nurse received a personalized 10-minute briefing to explain the purpose of the study and general instructions for completing the questionnaires. Nurses were advised to read each statement carefully and choose the response option that reflected their point of view. Each participant took 15–20 min to complete the questionnaires. The data collection period lasted three months, from early August 2024 to the end of October 2024.

### Statistical analysis

The sociodemographic and work-related data were summarized in terms of frequency and percentages. Continuous variables are displayed as the means, standard deviations (SDs), and mean percent scores. All the statistical analyses were performed via IBM SPSS Statistics version 23 (Armonk, NY). To examine the relationships between the study variables, Pearson’s correlation coefficient was used to assess the associations between normally distributed quantitative variables. The significance of the correlation coefficient was evaluated at *p* ≤ 0.05 and used for all the statistical tests. Furthermore, hierarchical regression analysis was conducted to explore the degree of variation in incident reporting culture among critical care nurses as influenced and predicted by leadership coaching behavior while controlling for demographic and work-related factors.

## Results

### Socio-demographic and professional characteristics of the studied nurses

As shown in Table 1, approximately three-quarters (73.3%) of the study subjects were female. More than one-half (53.3%) of the study participants were < 30 years old. In terms of marital status, the data revealed that more than two-fifths of the participants were married (47.9%). With respect to educational qualifications, more than two-fifths (45%) of the study subjects held bachelor’s degrees in nursing. Furthermore, regarding nursing experience, more than one-third (37.5%) of the subjects had less than five years of experience. Finally, the data on experience in the current unit indicated that over half (51.7%) had been working there for less than five years. A total of 74.6% of the study participants had never attended training programs related to different patterns, including coaching leader behavior, and the majority of the study participants (84.2%) had never attended training programs related to different patterns, including the reporting of expected incidents.

**Table 1:** Distribution of the participants according to demographics and professional characteristics (*n* = 240)

Demographics and professional characteristics	No.	%
**Sex**		
Male	64	26.7
Female	176	73.3
**Age**		
< 30	128	53.3
31–40	73	30.4
41–50	26	10.8
> 50	13	5.4
**Marital Status**		
Married	115	47.9
Single	107	44.6
Divorced	12	5.0
Widowed	6	2.5
**Education level**		
Nursing high school diploma	63	26.3
Technical Nursing institute diploma	69	28.8
Bachelor of nursing science	108	45.0
**Years of nursing experience in nursing profession**		
< 5	90	37.5
5–10	68	28.3
11–20	25	10.4
> 20	57	23.8
**Years of experience in the current unit**		
< 5	124	51.7
5–10	47	19.6
11–20	20	8.3
> 20	49	20.4
**Have you ever attended training program related to different patterns including leader coaching behavior?**		
Yes	61	25.4
No	179	74.6
**Have you ever attended a training program that Includes reporting of expected incidents?**		
Yes	38	15.8
No	202	84.2

### Descriptive statistics of the study variables

Table 2 shows that most of the study participants had moderate perceptions regarding their leadership coaching behavior and incident reporting culture (66.18% and 55.69%, respectively). The overall score (mean score ± standard deviation) of leadership coaching behavior (possible score range: 1–5) was 3.65 ± 0.82, and giving and receiving feedback had the highest mean domain score (3.79 ± 0.86). The overall mean score of incident reporting culture (possible score range: 1–5) was 3.23 ± 0.50, and the application of learning from errors had the highest mean score (3.51 ± 0.65).

### Correlations of the study variables

Table 3 reveals a moderately positive statistically significant correlation between nurses’ perceptions of incident reporting culture and leader coaching behavior (*r* = 0.617 & *p* < 0.001^*),^ and there was a statistically significant difference between the dimensions of the variables.

### Hierarchical multiple linear regression analysis

Hierarchical multiple linear regression analysis was conducted to examine the factors influencing incident reporting culture among the studied nurses (Table 4). In Model 1, demographic and professional characteristics (gender, age, marital status, educational qualifications, years of nursing experience, and years of experience in the current unit) collectively accounted for 23.5% of the variance in incident reporting culture (R² = 0.235, adjusted R² = 0.175, F = 2.268, *p* = 0.038). Within this model, age (β = 0.240, *p* = 0.025) and educational qualifications (β = -0.230, *p* = 0.024) emerged as significant predictors.

In Model 2, the variable Leader Coaching Behavior was entered, resulting in a statistically significant improvement in model fit. The explained variance increased from 23.5 to 50.5% (R² = 0.505, adjusted R² = 0.477, F = 22.168, *p* < 0.001), with an additional 27.0% of the variance explained (ΔR² = 0.270, *p* < 0.001). In this final model, leader coaching behavior was the strongest predictor of incident reporting culture (β = 0.608, *p* < 0.001), whereas the effects of age and educational qualifications were no longer statistically significant. These findings indicate that leader coaching behavior substantially enhances the explanatory power of the model and plays a pivotal role in shaping a positive incident reporting culture in critical care nursing.

**Table 2:** Descriptive statistics of the study variables among nurses (*n* = 240)

Items	Mean ± SD	Mean percent score %
Communication	3.71 ± 0.91	67.65 ± 22.72
Give and receive feedback	3.79 ± 0.86	69.65 ± 21.49
Delegate power and exert influence	3.51 ± 0.96	62.71 ± 23.96
Support the team to achieve organizational results	3.59 ± 0.95	64.71 ± 23.84
**Overall Leader Coaching Behavior**	**3.65 ± 0.82**	**66.18 ± 20.47**
Application of learning from errors	3.51 ± 0.65	62.77 ± 16.34
Readiness to provide feedback on incident reports	3.50 ± 0.68	62.40 ± 16.89
Collegial atmospheres of unpleasantness and punishment	2.72 ± 0.80	43.02 ± 19.88
Confidential and system driven	3.11 ± 0.83	52.63 ± 20.73
**Overall Incident Reporting Culture**	**3.23 ± 0.50**	**55.69 ± 12.60**

The perception levels were classified on the basis of the percentage of the maximum possible score as follows: low (< 50%), moderate (50–75%), and high (> 75%)

**Table 3:** Correlations between study variables (*n* = 240)

Leader Coaching Behavior	Incident reporting culture									
	Application of learning from errors		Readiness to provide feedback on incident reports		Collegial atmospheres of unpleasantness and punishment		Confidential and system driven		Overall	
	*r*	*p*	*r*	*p*	*r*	*p*	*r*	*p*	*r*	*p*
Communication	0.520	< 0.001^*^	0.437	< 0.001^*^	0.168	0.009^*^	0.266	< 0.001^*^	0.498	< 0.001^*^
Give and receive feedback	0.510	< 0.001^*^	0.385	< 0.001^*^	0.204	0.001^*^	0.277	< 0.001^*^	0.492	< 0.001^*^
Delegate power and exert influence	0.596	< 0.001^*^	0.547	< 0.001^*^	0.196	0.002^*^	0.431	< 0.001^*^	0.633	< 0.001^*^
Support the team to achieve organizational results	0.543	< 0.001^*^	0.502	< 0.001^*^	0.167	0.010^*^	0.368	< 0.001^*^	0.565	< 0.001^*^
**Overall**	0.611	< 0.001^*^	0.528	< 0.001^*^	0.206	0.001^*^	0.380	< 0.001^*^	0.617	< 0.001^*^

r: Pearson coefficient

*: Statistically significant at *p* ≤ 0.05

**Table 4:** Hierarchical multiple linear regression analysis to assess factors affecting the incident reporting culture of the studied nurses

	Step 1 (Model 1)					Step 2 (Model 2)				
	B	SE	β	t	*p*	B	SE	β	t	*p*
(Constant)	70.519	5.601		12.591	< 0.001^*^	37.973	5.282		7.190	< 0.001^*^
Gender	-1.252	1.572	-0.055	-0.796	0.427	1.072	1.271	0.047	0.843	0.400
Age	2.776	1.234	0.240	2.250	0.025^*^	1.785	0.989	0.155	1.805	0.072
Marital status	1.099	1.537	0.055	0.715	0.475	0.415	1.228	0.021	0.338	0.736
Educational qualifications	-2.806	1.235	-0.230	-2.272	0.024^*^	-1.245	0.995	-0.102	-1.252	0.212
Years of nursing experience	0.278	1.369	0.033	0.203	0.839	0.222	1.093	0.026	0.203	0.839
Years of experience in the current unit	-2.337	1.302	-0.277	-1.794	0.074	-2.014	1.040	-0.238	-1.937	0.054
Leader Coaching Behavior						0.374	0.032	0.608	11.568	< 0.001^*^
Model Summary:	Model 1: *R* = 0.485, R² = 0.235, Adjusted R² = 0.175, F = 2.268, *p* = 0.038* Model 2: *R* = 0.710, R² = 0.505, Adjusted R² = 0.477, F = 22.168, *p* < 0.001* ΔR² = 0.270**, *p* < 0.001* (additional variance explained by Leader Coaching Behavior)									

B: Unstandardized coefficients, β: standardized coefficients, t: t test of significance, R2: coefficient of determination, SE: standard error, t: t test of significance *: p is statistically significant at the 0.05 level (2-tailed)

## Discussion

This study investigated the level of leadership coaching behavior and incident reporting culture among CCNs. The first key finding indicated a moderate perception level regarding their leadership coaching behavior and incident reporting culture (66.18% and 56.69%, respectively). These findings suggest that nurses with direct patient care responsibilities perceive their leadership coaching behaviors as only moderately effective, which may influence their willingness to report incidents. The relatively lower percentage for incident reporting culture (56.69%) indicates potential barriers in creating an environment where nurses feel safe in reporting errors without fear of punishment. Additionally, nurses with moderate perceptions may recognize the importance of leadership behaviors and patient safety practices but may lack the necessary support, training, or systemic encouragement to fully implement them. Furthermore, these findings are consistent with those of Labrague & Obeidat (2022), who highlighted that transformational leadership styles, including coaching behaviors, are associated with improved communication, safety climates, and staff error reporting and empowerment in acute care settings [31].

These findings are further supported by recent literature. For example, Guerra-Paiva et al. (2023) and Yusuf & Irwan (2021) identified coaching behavior and transformational leadership as key facilitators of psychological safety and effective communication, both of which are essential for incident reporting [32, 33]. El-Sayed et al. (2022) and Adikoeswanto et al. (2024) also emphasized the direct role of supportive leadership in enhancing reporting behaviors [13, 34]. However, other studies, such as Fekadu et al. (2025), highlight that leadership alone may not suffice unless complemented by system-level reforms that address cultural and structural barriers. This suggests that while leadership coaching behavior is necessary, it must be integrated into a broader organizational strategy for patient safety [35].

The second finding demonstrated a positive association between nurses’ perceptions of incident reporting culture and leader coaching behavior. This suggests that when nurses perceive their leaders as engaging in supportive coaching practices such as providing feedback, encouraging open communication, and guiding professional development, they are more likely to view the incident reporting culture as positive and nonpunitive. Recent studies support this interpretation. For example, Knowles (2025) reported that leadership coaching behaviors, particularly those that involve individualized consideration and intellectual stimulation, significantly increase nurses’ willingness to report errors by fostering a psychologically safe environment [36]. Similarly, a study by Montminy (2022) highlighted that nurse managers who regularly coach and mentor their staff contribute to a culture where reporting incidents is seen as an opportunity for learning rather than punishment. These findings underscore the critical role of leadership in shaping safety culture, where coaching behaviors not only improve performance but also promote transparency and continuous improvement within healthcare teams. In essence, this positive association suggests that cultivating leaders who are effective coaches can be a powerful strategy for transforming an incident reporting culture into one that is truly open, learning-oriented, and ultimately safer for patients [37].

These findings are further supported by studies indicating that effective leadership practices, particularly those involving coaching and mentorship, play a pivotal role in strengthening safety cultures within healthcare settings. For example, Chegini et al. (2020) reported that nurses working under leaders who frequently engage in coaching conversations exhibit higher levels of trust and are more likely to participate in the voluntary reporting of adverse events [28]. Similarly, research by Lee et al. (2023) revealed that structured leader‒nurse interactions focused on reflective learning and error prevention significantly contribute to a more open and proactive reporting environment. These studies reinforce the idea that coaching behaviors from leaders can foster psychological safety, reduce fear of blame, and encourage a continuous learning environment, all of which are essential components of a robust incident reporting culture [38].

Nonetheless, some studies suggest that the impact of leader coaching behavior may vary depending on contextual factors such as high workload, staffing levels, and organizational hierarchy, cultural attitudes toward authority and accountability and resource constraints, which may moderate the effectiveness of leadership coaching. As supported by Aspinall et al. (2021), in settings with high workloads and limited managerial presence, the benefits of leader coaching on incident reporting are significantly diminished. This suggests that while leader coaching is a powerful tool, its effectiveness is influenced by broader organizational and systemic conditions and thus should be implemented as part of a comprehensive strategy to improve patient safety culture [39].

Additionally, in the present study, the majority of nurses (74.6%) reported no prior formal training in leadership coaching. This finding has important implications for the scalability and sustainability of coaching-based interventions aimed at enhancing incident reporting culture. Without foundational coaching skills, nurse leaders may struggle to effectively model and reinforce the behaviors required to foster psychological safety and open communication. To address this gap, healthcare organizations should consider implementing structured, organization-wide training programs that equip leaders with evidence-based coaching competencies. Such initiatives may not only improve the consistency and quality of coaching behaviors across units but also strengthen the long-term impact of interventions designed to promote a positive reporting culture.

The third finding revealed that leader coaching behavior significantly predicted incident reporting culture, and hierarchical regression revealed that adding leadership coaching behavior increased the explained variance from 23.5 to 50.5% (ΔR² = 0.270, *p* < 0.001), making it the strongest predictor. This underscores the substantial influence that leadership practices have on nurses’ willingness and ability to report incidents. When leaders actively engage in coaching behaviors such as providing constructive feedback, supporting problem solving, and fostering open communication, nurses are more likely to perceive a psychologically safe environment conducive to reporting errors. This finding aligns with the study by Chegini et al. (2020), which demonstrated a significant association between leader coaching behavior and nurses’ intention to report errors [28]. Their regression analysis indicated that leader coaching behavior (B = 0.172, *p* = 0.004) was a significant predictor of the intention to report errors, highlighting the critical role of supportive leadership in promoting a positive reporting culture.

Furthermore, a systematic review by Yusriawati and Irwan (2021) revealed that coaching leadership styles positively influence the culture of patient safety incident reporting. The review emphasized that coaching behaviors, characterized by guidance, support, and constructive feedback, are effective in encouraging healthcare staff to report incidents, thereby increasing patient safety [33]. These findings collectively highlight the pivotal role of leader coaching behaviors in shaping a robust incident reporting culture. Implementing leadership training programs that enhance coaching competencies could be instrumental in fostering an environment of learning and transparency, ultimately contributing to improved patient safety outcomes.

## Conclusion

The findings of the study demonstrate that leader coaching behavior significantly influences CCNs’ perceptions of reporting culture, accounting for a substantial portion of its variance. This suggests that when leaders consistently engage in supportive, feedback-driven, and development-focused interactions, critical care nurses are more likely to report incidents confidently and without fear of blame. Given the cross-sectional, single-site design, causality cannot be inferred, and further multisite or longitudinal research is needed.

### Implications and recommendations for practice

This study underscores the pivotal role of leader coaching behavior in fostering a positive incident reporting culture among critical care nurses. Nurse managers should adopt and consistently apply coaching-based leadership approaches characterized by active listening, constructive feedback, support for professional development, and facilitation of open dialog regarding errors to cultivate psychological safety and encourage transparent reporting without fear of blame or retribution.

Healthcare organizations should invest in targeted leadership development programs that build coaching competencies, particularly during nursing curricula and hospital onboarding. Embedding these competencies early in nurses’ careers enhances their long-term capacity to contribute to a robust safety culture. Additionally, systemic enablers such as allocating protected time for incident reporting, implementing user-friendly anonymous reporting systems, and establishing efficient feedback loops for reported incidents should be prioritized.

Institutional policies should explicitly recognize and reward coaching behaviors as integral to leadership performance. When combined with streamlined reporting processes and organizational support, these measures can strengthen transparency, accountability, and responsiveness within critical care settings. Ultimately, the integration of leadership coaching with systemic support offers a dual pathway to improving patient safety, reducing preventable harm, and enhancing the overall quality of care in high-pressure environments such as critical care units.

### Strengths and limitations of the study

This study addresses a critical gap in patient safety research by linking leadership coaching to incident reporting culture in critical care. It applies validated instruments (IRCQ and LCBQ), a sufficient sample size, and rigorous statistical analyses, grounded in psychological safety theory and the leadership coaching model, yielding both theoretical and practical implications for nursing leadership development and hospital policy.

This study has several limitations that should be acknowledged. The cross-sectional design limits the ability to determine causality between leader coaching behavior and incident reporting culture, and reliance on self-reported data may introduce bias. The study’s focus on a specific group of critical care nurses within a single setting also limits the generalizability of the findings.

Future studies should use longitudinal, experimental, or mixed-methods approaches to clarify causal links between leader coaching behavior and incident reporting culture. To enhance validity and generalizability, data should be drawn from multiple sources and settings involving diverse professional groups. Qualitative methods are also recommended to explore how coaching behaviors drive lasting cultural change.

## Data Availability

The datasets used and/or analyzed during the current study are available from the corresponding author upon reasonable request.

## Abbreviations

CCUs: Critical care units
CCNs: Critical care nurses
